# Factors influencing a health-promoting lifestyle in participants after undergoing repeat percutaneous coronary intervention based on the Capability, Opportunity, Motivation-Behaviour model: a qualitative study

**DOI:** 10.1186/s40359-026-03981-0

**Published:** 2026-01-12

**Authors:** Zhijie Cao, Fang Hou, Lina Ma, Xiaoyu Zhang, Xuefei Lu, Li Zhang

**Affiliations:** 1 Nursing School, Xinjiang Medical University, Urumqi, 830000 China; 2 Department of Nursing, The First Affiliated Hospital of Xinjiang Medical University,State Key Laboratory of Pathogenesis, Prevention and Treatment of High Incidence Diseases in Central Asia, No. 393, Liyue Mountain Avenue, Urumqi, 830000 China; 3 The First Affiliated Hospital of Xinjiang Medical University, Urumqi, 830000 China; 4 Health Care Research Center for Xinjiang Regional Population, Urumqi, 830000 China

**Keywords:** COM-B model, Post percutaneous coronary intervention, Health-promoting lifestyle, Influencing factors, Qualitative research

## Abstract

**Background:**

Coronary heart disease is a leading cause of global mortality, for which percutaneous coronary intervention serves as a critical revascularization strategy. Despite the recognized importance of health-promoting behaviors, participants face significant challenges in adopting and sustaining these behaviors post-procedurally. Current research predominantly focuses on primary PCI participants, leaving a gap in the systematic investigation of unique factors influencing health-promoting lifestyles specifically within the higher-risk population undergoing repeat PCI. This distinct subgroup often presents with a history of behavioral relapse, greater psychological distress, and altered risk perception, which may not be adequately addressed in standard care. Therefore, this study employs the (Capability, Opportunity, Motivation-Behaviour) model to elucidate the multifaceted barriers affecting the maintenance of a health-promoting lifestyle in this distinct participants cohort.

**Objective:**

To explore the factors influencing whether participants adopt a healthy lifestyle after repeat percutaneous coronary intervention, to provide healthcare professionals with a theoretical basis from which they can develop targeted intervention programmes.

**Methods:**

A descriptive phenomenological research method was used to develop an interview outline, using the Capability, Opportunity, Motivation-Behaviour model as a guide. Sixteen participants who underwent repeat coronary intervention were selected to participate in semi-structured interviews. Data were analysed using Colaizzi’s seven-step method to summarise and extract themes.

**Results:**

Capability-related factors included inaccurate knowledge of the disease, inadequate skills to manage medication, lack of physical strength and health limitations, and poor skills for psychological and emotional adjustment. Opportunity-related factors included insufficient family and environmental support, limited access to medical resources, conflicts with regional dietary habits, and the influence of work habits. Motivation-related factors included a willingness to proactively manage health, psychological and emotional regulation, behavioural relapse, and inertia.

**Conclusion:**

Multiple factors influence the adoption of health-promoting lifestyles in participants undergoing repeat percutaneous coronary intervention. Healthcare professionals need to consider several perspectives to improve participants’ knowledge of how to promote their health after surgery and reduce barriers to health-promoting behaviours. This will help improve the overall health of participants in this population.

## Introduction

Coronary heart disease (CHD) is one of the most common and lethal diseases worldwide [1]. It is also a leading cause of disability, placing a heavy burden on healthcare systems [2]. According to the Global Burden of Disease 2019, 9.14 million deaths worldwide are caused by CHD, accounting for 49.2% of all cardiovascular deaths and 16.3% of all-cause mortalities [3]. According to the China Health Statistics Yearbook 2022, the mortality rate of CHD among Chinese nationals was 135.08 per 100,000 in 2021 [4]. The prevalence of this disease will continue to rise in the next decade [5], making CHD one of the biggest public health issues in China.Percutaneous coronary intervention (PCI) is a technique in which cardiac catheterisation is used to open narrowed or occluded coronary arteries, thereby improving myocardial blood flow [6]. It has become a primary clinical treatment for CHD because its use offers advantages such as minimal invasiveness, short procedure time, and rapid recovery [7]. Although participants may achieve beneficial outcomes after stenting, it does not reverse or halt the progression of atherosclerosis [8]. The occurrence of adverse cardiovascular events has become a serious clinical problem [9], and in-stent restenosis after PCI has drawn increasing attention [10]. Currently, in-stent restenosis represents a popular research topic and significant challenge [8]. Notably, participants undergoing repeat PCI represent a distinct and higher-risk subgroup compared to those undergoing first-time PCI. They often present with more complex clinical conditions, including a heavier burden of comorbidities, longer exposure to cardiovascular risk factors, and a history of relapse of unhealthy behaviors. Having experienced a cardiac event despite prior interventions, these participants may also suffer from more intense psychological distress and altered perception of risk. However, current research predominantly focuses on primary PCI recipients, leaving a gap in understanding the unique factors influencing health-promoting lifestyles specifically within this higher-risk population undergoing repeat PCI [11].Therefore, this study focuses on the specific subgroup of repeat PCI participants, aiming to identify the unique barriers and facilitators affecting health-promoting behaviors that may have been insufficiently addressed in the broader population of PCI participants.

Lifestyle plays an important role in the development of CHD [12], with an unhealthy lifestyle being a key factor that contributes to poor prognosis, recurrence, and rehospitalisation after treatment. High body mass index, smoking, poor diet, low levels of physical activity, and mental health issues have all been found to be modifiable risk factors for cardiovascular disease [13]. Lifestyle changes should be the basis of managing CHD [14]. Adopting healthy lifestyle behaviours, including avoiding tobacco use and harmful alcohol intake as well as maintaining a healthy diet, ideal body weight, and physical activity, are the most cost-effective strategies for preventing CHD [15]. For participants who have undergone PCI, postoperatively engaging in health-promoting behaviours is essential for improving physical and psychological function, quality of life, and long-term outcomes. Engaging in such behaviours would achieve comprehensive prevention of cardiovascular disease and premature death [16].The Capability, Opportunity, Motivation-Behaviour (COM-B) model [17] can be used to analyse the mechanisms and influencing factors underlying the occurrence of target behaviours. It has been widely applied to manage chronic disease, promote health, and prevent disease [18].

Therefore, this study focuses on the specific and under-researched subgroup of repeat PCI participants. It employs the COM-B model as a theoretical framework and uses a descriptive phenomenological research method to conduct an in-depth analysis of the factors influencing health-promoting lifestyles in participants following repeat PCI. The aim was to provide a theoretical basis from which healthcare professionals could develop targeted interventions that they could use to encourage participants to value and consciously adopt healthier lifestyles after PCI.

## Methods

### Study design

The research team consisted of experienced qualitative researchers with backgrounds in nursing and psychology. All interviewers had prior training in qualitative methods and were familiar with the COM-B model. To minimize bias, the team reflected on their assumptions about participant behavior and maintained a neutral stance during data collection. Regular debriefing sessions were held to discuss potential biases and ensure consistency.

### Study participants

During this period, all eligible participants were invited to participate to ensure a diverse sample in terms of gender, age, and residence. The team aimed to include participants with varying demographics to capture a range of experiences. Inclusion criteria were as follows: (1) participants who met the diagnostic criteria of the Chinese Guidelines for Percutaneous Coronary Intervention (2016) [19] and had successfully undergone PCI more than once; (2) aged ≥ 18 years; (3) provided informed consent and volunteered to participate in the study. The exclusion criteria were (1) participants with cognitive or mental disorders, or functional impairments in vision or hearing; (2) participants with functional impairments in vital organs or comorbidities with serious chronic diseases of other systems; and (3) participants with speech disorders or communication difficulties. These criteria were assessed using medical records and clinical evaluations conducted by cardiologists and nurses. During the three-month recruitment period, a total of 28 eligible participants were identified. Of these, 16 agreed to participate. The remaining 12 participants declined, primarily due to discomfort with audio recording, lack of interest in research participation, or immediate post-discharge fatigue. The sample size was determined based on data saturation during interviews. Data saturation was considered achieved when no new themes or insights emerged from three consecutive interviews, which occurred after the 14th interview. The final two interviews confirmed the existing themes, ensuring thematic completeness. This study was approved by the Ethics Committee of the hospital. Participation was voluntary, and informed consent was obtained from all participants.

### Development of the interview guide

The interview guide was jointly developed by a team of experts consisting of cardiologists and nursing specialists. Prior to the formal interviews, three participants who met the inclusion and exclusion criteria were selected for pilot interviews. The guide was revised and refined based on the results of the pilot interviews, and the final interview content was confirmed. Although some questions were not fully open-ended, the interviewers guided the participants to provide detailed responses through probing, prompting, and other methods to ensure the collection of rich research data.

The outline of the interview was as follows: (1)After the first surgery, in what ways did you pay more attention to your health, and what behaviours did you adopt to promote your health? (2) Have you since stopped any of these behaviours? (3) What do you think caused you to have to undergo a second surgery? (4)Do you think these causes are related to your usual lifestyle habits? (5)Do you have any plans to improve your lifestyle in the future, and in what areas? (6) What do you intend to do to achieve these goals? See Table 1 for details.

**Table 1:** Interview questions and corresponding COM-B dimensions

Question Number	COM-B Dimension	Question
1	Capability	After the first surgery, in what ways did you pay more attention to your health, and what behaviours did you adopt to promote your health?
2	Capability	Have you since stopped any of these behaviours?
3	Opportunity	What do you think caused you to have to undergo a second surgery?
4	Opportunity	Do you think these causes are related to your usual lifestyle habits?
5	Motivation	Do you have any plans to improve your lifestyle in the future, and in what areas?
6	Motivation	What do you intend to do to achieve these goals༟

### Data collection

The study was based on a descriptive phenomenological approach, with data collected through semi-structured interviews. The researchers were professionally trained in qualitative research and had good communication and interviewing skills. The interviews were conducted in quiet private wards fitted with audio recording equipment. The interviews were carried out after informed consent was obtained, on condition that the participant’s mental state was stable and the interview would not interfere with medical treatment. Prior to starting the interview, each participant was informed of the study purpose, the need to record the interview, and confidentiality measures (anonymisation via digital coding). Participants were assured that they could terminate or reschedule the interview at any time. Mock interviews were conducted in advance among the research team to standardise procedures. A second team member was present during each interview to assist in capturing non-verbal cues such as body language and tone. Each session lasted approximately 45 to 60 min. Interviewers employed questioning, active listening, and follow-up prompts to create a relaxed atmosphere and encourage participants to share genuine experiences. Interviews concluded once data saturation was reached and no new themes emerged.

### Data processing and analysis

The audio recordings were transcribed by the interviewer within 24 h of the interview and checked for accuracy by another member of the team. The transcribed texts were then imported into NVivo 12.0 for organisation and thematic analysis. The analysis followed Colaizzi’s seven-step method [20]: All transcripts were thoroughly read to gain a general understanding; significant statements were extracted; meanings were formulated from these statements; meanings were organised into themes and categories based on commonalities; themes were integrated into a comprehensive description of the phenomenon; an essential structure of the experience was formulated; and the findings were validated by giving them back to the participants for confirmation.The COM-B model served as the core analytical framework throughout this process. Specifically, initial coding was conducted inductively based on the interview content to identify recurring and meaningful insights. Subsequently, these inductively derived codes and themes were mapped onto and structured within the three principal domains of the COM-B model: capability, opportunity, and motivation. The use of a theoretical framework within a phenomenological approach allowed for a structured understanding of participants’ lived experiences while preserving the richness of their narratives. Data analysis was conducted by two researchers (CZJ and MLN) independently, and any discrepancies were resolved through discussion with the team.

## Results

### General information of respondents, interview themes and citations

A total of 16 respondents were included in this study, and their basic information is shown in Table 2. The results of the interviews were analysed and summarised based on the COM-B model to extract the factors that influence the adoption by participants of health-promoting lifestyles after repeat PCI. Ultimately, 6 themes and 11 sub-themes were analysed (Table 3).

**Table 2:** General information about respondents (*n* = 16)

	Number	Percentage (%)
Sex		
Male	12	75.00
Female	4	25.00
Age		
18–34	0	0
35–49	2	12.5
50–65	5	31.25
≥ 65	9	56.25
Education level		
Lower secondary education or below	2	12.5
Upper secondary education or vocational qualifications	8	50.00
Further education diploma	1	6.25
Undergraduate degree and above	5	31.25
Marital status		
Single	0	0
Married	15	93.75
Divorced	0	0
Widowed	1	6.25
Monthly household income		
< 1000	1	6.25
1000–3000	1	6.25
3001–5000	3	18.75
≥ 5001	11	68.75
Employment status		
Employed	9	56.25
Retired	3	18.75
Unemployed	4	25.00
Smoking status		
Yes	1	6.25
No	15	93.75
Alcohol consumption		
Yes	1	6.25
No	15	93.75
Overweight status		
Yes	9	56.25
No	7	43.75
First-time diagnosis of coronary heart disease		
Yes	3	18.75
No	13	81.25
Disease duration		
< 5 years	12	75.00
≥ 5 years	4	25.00
Presence of comorbidities		
Yes	16	100.00
No	0	0

**Table 3:** Themes and quotes for factors influencing health-promoting lifestyles after repeat percutaneous coronary intervention based on the Capability, Opportunity, Motivation-Behaviour model

COM-B model aspect	Theme	Subtheme	Illustrative quotations
**Capability**	Insufficient knowledge and skills	Misconceptions about disease	P1: ‘I do not believe it’s 100% blocked. They might have made a mistake, because I did not really feel anything, just some tightness in the throat after eating, but no pain or pressure like others say …’ P8: ‘After the last stent, I did not pay much attention. This time it was back pain. I thought it was gallbladder inflammation. I took some medicine and felt a bit better, but still uncomfortable, so I came back …’ P10: ‘What counts as moderate exercise? Can I swim? Is lifting three kilograms okay? …’ P11: ‘Usually, I do not care about anything, this time I came to accompany my daughter to the doctor and stopped by to check …’
		Inadequate management of medication	P7: ‘At last year’s follow-up, they said I had some gastrointestinal bleeding, probably from long-term use of Plavix and enteric-coated aspirin, so I stopped taking both and never resumed …’
	Limited physical capability	Physical and health status limitations	P1: ‘I cannot walk fast, only slowly, and I do not usually jump rope or jog …’ P9: ‘I am going to have to exercise less and be less physically active this time than I was before because I am afraid I am going to have chest pains from exercising …’
		Lack of skills for psychological and emotional adjustment	P3: ‘I am scared. I am scared that if I exercise, my stent will come off, so I exercise less …’ P6: ‘I have a bit of a bad personality and get angry easily …’ P8: ‘After my back hurt, I wondered if it was blocked. I could not sleep at night, just thinking, do not die, do not die …’ P13: ‘I am grumpy, not very emotionally stable, and cannot control it …’
**Opportunity**	Family and environmental opportunities	Insufficient family and environmental support	P6: ‘I take care of everything at home, I do all the labour in the house, I do all the cooking and cleaning, I do all the farming, I probably just labour too much and do too much heavy lifting …’ P9: ‘It might have something to do with genetics too, both my grandparents died of coronary heart disease …’
		Inadequate access to medical resources	P3: ‘The local hospital is not very good, so I only feel comfortable coming to a big hospital, but it is inconvenient, that is why I did not follow up…’ P6: ‘I was supposed to have surgery for hydronephrosis, but my blood pressure was too high. Since I had a stent before, the local doctor told me to come here and check the heart first …’ P14: ‘I have studied a lot about the heart’s structure, blood vessels, and treatment options on my own …’
	Sociocultural opportunities	Conflicts with regional dietary habits	P1: ‘Especially when it comes to eating, there has been no change in my palate either—I still heavy on salt, flavour, and spice …’ P10: ‘It is all about lamb, beef, and horse meat here in Xinjiang, we eat it like crazy …’ P12: ‘I love meat so much … How can Xinjiang people live without it? …’
		Influence of work habits	P2: ‘I am busy because of work and I am on duty at night, so I drive everywhere I go …’ P10: ‘I also used to have to drink and socialise for work …’ P15: ‘The nature of my job involves night shifts, so my routine is not really very regular …’
**Motivational factors**	Self-motivated	Willingness to proactively manage health	P4: ‘I was walking regularly, about 8,000 to 10,000 steps a day …’ P5: ‘I try to eat less meat and offal, and stick to lighter foods as much as possible …’ P12: ‘No smoking or drinking. I have given up all of those …’
		Psychological and emotional adjustment	P10: ‘There is an old Chinese saying: “At fifty, one accepts fate”. Just go with the flow. Stay calm and accept whatever happens …’ P13: ‘I am going to control my temper and emotions through meditation, or be less angry; emotions are important too, I am grumpy, not very stable or controlled, but I need to keep a happy mood …’
	Motivation for reflection	Behavioural relapse and inertia	P3: ‘… It felt like a hassle, so I did not go for a follow-up …’ P7: ‘My lifestyle habits have become bad again. Smoking again, smoking and eating, staying up late …’ P13: ‘I did not stick to my diet, and gradually gained back the weight …’ P16: ‘After the surgery, I did not feel anything, no symptoms at all, so I did not go for follow-up …’

*COM-B* Capability, Opportunity, Motivation-Behaviour, *P* Participant

### Capability factors: dual limitations of physical-mental capabilities and knowledge-skills

#### Insufficiency of knowledge and skills

In this subtheme, we found that participants exhibited deviations in disease cognition and weaknesses in medication management capabilities. Most participants lacked a correct understanding of the core knowledge regarding coronary heart disease and the post-percutaneous coronary intervention period. Their cognition was mostly derived from passive event-triggered exposure rather than active learning. Such limitations and lag in cognition directly led to the absence or deviation of their health management behaviors. Some participants delayed seeking medical treatment due to underestimating their condition or making incorrect attributions for their symptoms; others arbitrarily discontinue medications out of fear of side effects. Consequently, they failed to develop systematic, continuous, and scientific postoperative self-management behaviors.

P1: ‘I do not believe it’s 100% blocked. They might have made a mistake, because I did not really feel anything, just some tightness in the throat after eating, but no pain or pressure like others say. If I don’t walk after meals, I feel perfectly fine. Symptoms like pain, stuffiness, pain during physical activity, and excessive sweating that others have never happen to me.’

P11: ‘It has been five years since my first surgery, and I haven’t had any reexaminations during this period. I didn’t pay much attention to my health on a daily basis. I came here this time to accompany my daughter for her medical check - up and decided to take a simple examination by the way. I don’t do excessive physical exercise, don’t feel any pain, and have a normal appetite.’

P7: ‘At last year’s follow-up, they said I had some gastrointestinal bleeding, probably from long-term use of Plavix and enteric-coated aspirin, so I stopped taking both and never resumed.’

#### Impairment of physical execution capabilities

This subtheme highlighted that participants faced multiple challenges related to physical function, cognitive psychology, and emotional management. A clear interaction emerged between physical limitations and psychological–emotional difficulties. For instance, reduced physical activity was not solely due to functional decline but was compounded by fear of exercise-related risks and poor emotional regulation, which together undermined confidence and recovery. The reduction in their physical activity stemmed not only from actual declines in physical function but also from the combined constraints of excessive fear of exercise-related risks and vague understanding of appropriate exercise methods, prompting participants to adopt activity-avoidance strategies. Meanwhile, participants struggled with emotional regulation. A tendency toward irritability intertwined with intense anxiety about disease progression seriously undermined their psychological stability and confidence in recovery. This indicated that psychological support and exercise guidance play equally crucial roles in postoperative management.

P9: ‘I cannot walk fast, only slowly, and I do not usually jump rope or jog. My spouse can easily leave me far behind while walking.’

P8: ‘After my back hurt, I wondered if it was blocked. I could not sleep at night, just thinking, do not die, do not die. On my way to the hospital, I couldn’t stop worrying and fearing that the artery was blocked once more.’

### Opportunity factors: lack of external support and environmental resources

#### Family and environmental opportunities

Here, we identified that participants lacked effective family support and accessible medical resources. The heavy burden of housework made it difficult for them to rest adequately, and the inadequacy of primary medical resources had become a key barrier to their adherence to regular reexaminations. The systemic lack of such a supportive environment reflected the insufficiency of the postoperative health support system.

P6: ‘I take care of everything at home, I do all the labour in the house, I do all the cooking and cleaning, I do all the farming, I probably just labour too much and do too much heavy lifting. I was supposed to have surgery for hydronephrosis, but my blood pressure was too high. Since I had a stent before, the local doctor told me to come here and check the heart first. If everything is normal, we planned to proceed with the hydronephrosis surgery. However, the examination results showed that another stent needs to be implanted.’

P14: ‘I have studied a lot about the heart’s structure, blood vessels, and treatment options on my own.’

#### Socio - cultural opportunities

This subtheme indicated that the adoption of healthy lifestyles by participants was strongly constrained by both local cultural factors and occupational environments. Participants found it hard to change the long- established local dietary habits characterized by high salt and high fat content. Their reliance on meat intake and taste preferences posed major obstacles to dietary adjustments. Meanwhile, the irregular work schedules, reduced physical activity, and unavoidable social drinking resulting from the nature of their occupations further limited their ability and opportunities to practice healthy behaviors. Such structural barriers deeply rooted in daily life and occupational settings weakened the feasibility of health-promoting behaviors. This suggested that personalized health guidance can only be effective when combined with extensive environmental support.

P1: ‘Especially when it comes to diet, I haven’t changed my taste at all. I still prefer food that is high in salt, strong in flavor, and spicy. I’m quite stubborn about my eating habits.’

P10: ‘The meat here is so delicious! It is all about lamb, beef, and horse meat here in Xinjiang, we eat it like crazy.’

P15: ‘The nature of my job involves night shifts, so my routine is not really very regular. Sometimes I have to work day shifts before I fully recover from the night shifts.’

### Motivational factors

This theme captured the duality and instability in participants’ motivation to maintain healthy behaviors. Some participants could take the initiative to adopt and maintain healthy behaviors, which was reflected in regular exercise, dietary control, and quitting smoking and drinking. They also maintained emotional stability through psychological adjustment strategies. However, other participants exhibited unsustainable healthy behaviors. They lowered their guard when their symptoms eased, or their unhealthy behaviors rebounded due to laziness, external inconveniences, and other factors.

P4: ‘I was walking regularly, about 8,000 to 10,000 steps a day. I eat more whole grains for lunch and dinner.’

P5: ‘I try to eat less meat and offal, and stick to lighter foods as much as possible.I also stopped drinking alcohol completely after the first surgery.’

P13: ‘I am going to control my temper and emotions through meditation, or be less angry; emotions are important too, I am grumpy, not very stable or controlled, but I need to keep a happy mood and pay more attention to health preservation.’ ‘I did not stick to my diet, and gradually gained back the weight, though it’s still a bit lower than before.’

P16: ‘After the surgery, I did not feel anything, no symptoms at all, so I did not go for follow-up. Since I don’t feel any pain or discomfort, I don’t see the point in getting those checks done.’

## Discussion

### Enhancing physical, mental, and cognitive abilities as the foundation for promoting health in participants after PCI

Different types of rehabilitation exercises are safe and effective in reducing the incidence of coronary artery restenosis in participants [21]. Appropriate rehabilitation exercise contributes to the development of collateral circulation, minimises myocardial necrosis, and lowers mortality among participants with myocardial infarction [22]. Despite the benefits that participants gain from exercise rehabilitation after PCI, participation in such exercise is low, with fear of physical activity identified as a key influencing factor [23]. Common negative emotions such as anxiety and depression can adversely affect postoperative recovery [24]. Improving cognitive capability is key to overcoming barriers to healthy behaviours. By enhancing participants’ understanding of their symptoms, disease progression, and the role of medication and diet, the development of accurate health beliefs can be supported [25]. Therefore, targeted exercise guidance and education should be provided to participants after PCI to help them correctly assess their physical capacity, increase their knowledge of physical activity, and boost their confidence in engaging in exercise. This would help correct negative attitudes towards exercise and support the establishment of healthy behaviours. Furthermore, active psychological adjustment is essential. Psychological capability should be strengthened by offering participants guidance on managing their emotional and mental health [26]. Approaches such as mindfulness therapy, cognitive behavioural therapy, and meditation [27–29] can be used to support participants to recognise and appropriately manage their psychological states, thereby improving psychological resilience. By making healthy physical activity, positive emotional regulation, and accurate disease-related cognition a routine part of daily life, participants can develop greater awareness and autonomy after PCI, thus entrenching health-promoting behaviours into their everyday lifestyle. The interplay between cognitive misinterpretation of symptoms and motivational disengagement observed in our participants reflects a broader behavioral pattern previously described as “behavioral delay” in post-PCI patients [30]. From a practical standpoint, our findings suggest that nursing and cardiac rehabilitation teams should prioritize structured educational programs and counseling aimed at improving participants’ capability (Capability domain). This includes clear instruction on medication management, tailored exercise prescriptions to build confidence, and psychological support techniques to address fear and anxiety, which are core components of cardiac rehabilitation.

### Enhancing opportunities and environmental support as an important way to improve health promotion for participants after PCI

The findings of this study revealed that participants experienced barriers related to family dietary habits, traditional regional cuisines, burdensome household responsibilities, work routines, and limited access to healthcare resources. These findings highlight the profound impact that opportunities and environmental factors have on health behaviours. Optimising opportunities and the broader environment to promote health emerged as a vital means of improving adherence to behaviours and prognosis in participants after PCI. The barriers that participants face in terms of their health behaviours stem from individual factors, as well as multiple constraints in the family, community, healthcare system, and cultural environments. A healthy family environment has been shown to improve disease management outcomes in participants with chronic illnesses, whereas a supportive community environment also plays a facilitating role [31]. Improving the level of health in the household can raise the health literacy of a participant with a chronic disease, encourage healthier behaviours, and contribute to better disease control [32]. Previous studies have demonstrated that a tripartite hospital–community–family intervention model can enhance adherence, improve cardiac function, and raise the quality of life of older participants after PCI [33]. Therefore, the opportunity domain can be optimized through a series of specific organizational interventions. Efforts should be made to promote the family’s engagement in health education, use community activities to enhance familial support, and strengthen collaborative healthcare networks and telemedicine coverage for participants in remote areas. In particular, home-based cardiac telerehabilitation, as a promising model of transitional care, has demonstrated efficacy in improving physical function, medication adherence, and health-related quality of life for post-PCI participants, while overcoming geographical and resource barriers [34].This requires broadening opportunities at the household and community levels while optimising the healthcare environment. Moreover, creating health-conscious versions of local dishes and disseminating disease-related knowledge and information on prognosis through various channels can increase access to health education and provide participants with more opportunities and supportive environments to promote their health [35].

### Strengthening motivation to promote health as a key element in improving the prognosis of participants after PCI

In this study, we found that although some participants were able to actively adjust their health behaviours, overall, they exhibited insufficient motivation to promote their health. Specific issues included inconsistent behavioural adherence, inertia in pursuing health goals, and a passive attitude towards risk perception. Similar patterns have been described in post-PCI populations, where symptom relief fosters a false sense of recovery and leads to behavioral delays in seeking care and reduced adherence to secondary prevention strategies [30]. This pattern was particularly salient in our cohort. A key theme emerging from our interviews was that the absence of acute symptoms after PCI often led participants to underestimate their ongoing cardiovascular risk. This diminished perception of risk, in turn, eroded their motivation for sustained vigilance and became a primary driver for the relapse of unhealthy behaviors, such as abandoning dietary controls, skipping medications, or discontinuing follow-up visits. The root cause of the lack of motivation can be attributed to the lack of intrinsic drive and the weakness of external support systems. Motivation is a core concept of behavioural change [36], and its sustainability depends on the interaction between autonomy, competence, and a sense of relatedness. In the present study, some participants lacked the internal desire that drives changes in behaviour, resulting in poor sustainability and frequent behavioural relapse or inertia. Change in behaviour that is driven by intrinsic motivation is more likely to lead to a healthy lifestyle being adopted and maintained [37]. Enhancing participants’ cognition and motivation is therefore crucial to improving their health behaviours. Intrinsic motivation is core, and optimising extrinsic motivation requires multidimensional synergy. Activating intrinsic motivation is essential, whereas optimising extrinsic motivation requires coordination on multiple levels [38]. Studies have shown that health literacy in a participant positively predicts medication adherence [39]. When participants believe they can improve their health through their actions, their levels of motivation increase [40]. Therefore, strengthening the participant’s awareness of the association between behaviour and outcome is necessary, as is helping the participant to establish the belief that behavioural change can slow down the progression of the disease. Addressing issues such as non-adherence, negative emotions, behavioural relapse, and inertia—which often stem from insufficient understanding of the disease—requires guiding participants towards accurate knowledge of their condition. This, in turn, can foster intrinsic motivation for behavioural change, support the establishment of scientifically informed lifestyles, and further reinforce the adoption of health-promoting behaviours. Clinically, this translates to integrating behavior change strategies within the Motivation domain, such as motivational interviewing, collaborative goal-setting, and health coaching into routine post-PCI care. These approaches can help participants internalize health goals, build self-efficacy, and sustain long-term behavior change.

### Integrating cultural and contextual considerations

The characteristics observed in this study, such as a significant preference for meat-rich diets, prominent impacts of occupational schedules, and limited accessibility to healthcare resources, are all closely associated with sociocultural and regional contexts. While these factors may restrict the direct generalizability of the study’s findings to other regions, the conclusions can provide actionable insights for designing culturally adaptive intervention programs in areas with similar dietary traditions, urban-rural development gaps, or occupational constraints. For instance, intervention efforts could prioritize strengthening community support, tailoring dietary guidance to local culinary cultures, and leveraging telemedicine to mitigate deficiencies in healthcare resource accessibility.

### Comparing with first-time PCI patients and deriving theoretical implications

While some barriers identified here—such as fear of exercise or dietary challenges—are also reported among first-time PCI patients, our findings highlight their heightened intensity and interconnectedness in the repeat-PCI cohort. For example, the relapse of unhealthy behaviors was not merely a failure of adherence but was often rooted in a diminished perception of risk following symptom relief, a phenomenon less pronounced in primary PCI patients who are often in a more vigilant “acute threat” phase. This reinforces the need for theory-driven, tailored interventions that address not only knowledge and skills but also the motivational and environmental reinforcers of behavior, especially in patients with a history of recurrent events.

## Limitations

All the interviewees in this study were participants at a tertiary hospital in Xinjiang; therefore, the risk of sample selection bias exists. Additionally, the cross-sectional nature of our interviews limits insights into how factors influencing health behaviors evolve over time. The potential for social desirability bias in self-reported behaviors should also be acknowledged, although our use of probing and neutral questioning aimed to mitigate this. Future research that includes participants from a broader range of regions and healthcare settings, and that adopts a mixed-methods approach, is recommended to provide more comprehensive evidence for promoting healthy lifestyles in participants after PCI.

## Conclusions

In this study, based on the COM-B model, we conducted in-depth interviews with 16 participants who had undergone repeat PCI. From the three dimensions of capability, opportunity, and motivation, six themes and eleven subthemes that influence the adoption of health-promoting lifestyles following PCI were identified. Our findings suggest that standardized, one-dimensional interventions are often insufficient for this complex population. Instead, an integrated approach that simultaneously addresses capability gaps, enhances environmental opportunities, and strengthens intrinsic motivation is needed to support sustained behavioral change. Healthcare professionals should therefore adopt a multidimensional perspective that considers the participants’ capabilities, environmental opportunities, and motivational factors in a holistic manner. Efforts should be made to enhance participants’ awareness of health promotion after PCI, value supportive health environments, foster intrinsic motivation, and reduce barriers to health-promoting behaviours, with the goal ultimately being to improve the overall health and quality of life of participants after PCI.

## Data Availability

The datasets used and/or analysed during the current study are available from the corresponding author on reasonable request.

## Abbreviations

CHD: Coronary heart disease
PCI: Percutaneous coronary intervention
COM-B: Capability, Opportunity, Motivation-Behaviour model
